# Dissecting the Epigenetic Changes Induced by Non-Antipsychotic Mood Stabilizers on Schizophrenia and Affective Disorders: A Systematic Review

**DOI:** 10.3389/fphar.2020.00467

**Published:** 2020-04-22

**Authors:** Manuel Gardea-Resendez, Mehmet Utku Kucuker, Caren J. Blacker, Ada M.-C. Ho, Paul E. Croarkin, Mark A. Frye, Marin Veldic

**Affiliations:** ^1^Department of Psychiatry, Universidad Autónoma de Nuevo León, Monterrey, México; ^2^Department of Psychiatry and Psychology, Mayo Clinic Depression Center, Mayo Clinic, Rochester, MN, United States

**Keywords:** epigenetics, mood disorders, schizophrenia, lithium carbonate, valproic acid, anticonvulsants, histone deacetylase inhibitors, DNA methylation

## Abstract

**Background:**

Epimutations secondary to gene-environment interactions have a key role in the pathophysiology of major psychiatric disorders. *In vivo* and *in vitro* evidence suggest that mood stabilizers can potentially reverse epigenetic deregulations found in patients with schizophrenia or mood disorders through mechanisms that are not yet fully understood. However, their activity on epigenetic processes has made them a research target for therapeutic approaches.

**Methods:**

We conducted a comprehensive literature search of PubMed and EMBASE for studies investigating the specific epigenetic changes induced by non-antipsychotic mood stabilizers (valproate, lithium, lamotrigine, and carbamazepine) in animal models, human cell lines, or patients with schizophrenia, bipolar disorder, or major depressive disorder. Each paper was reviewed for the nature of research, the species and tissue examined, sample size, mood stabilizer, targeted gene, epigenetic changes found, and associated psychiatric disorder. Every article was appraised for quality using a modified published process and those who met a quality score of moderate or high were included.

**Results:**

A total of 2,429 records were identified; 1,956 records remained after duplicates were removed and were screened *via* title, abstract and keywords; 129 records were selected for full-text screening and a remaining of 38 articles were included in the qualitative synthesis. Valproate and lithium were found to induce broader epigenetic changes through different mechanisms, mainly DNA demethylation and histones acetylation. There was less literature and hence smaller effects attributable to lamotrigine and carbamazepine could be associated overall with the small number of studies on these agents. Findings were congruent across sample types.

**Conclusions:**

An advanced understanding of the specific epigenetic changes induced by classic mood stabilizers in patients with major psychiatric disorders will facilitate personalized interventions. Further related drug discovery should target the induction of selective chromatin remodeling and gene-specific expression effects.

## Introduction

### Rationale

Psychiatric illness entails a severe disease burden that is associated with reduced productivity, quality of life, and life expectancy (Vigo et al., 2016). Globally, bipolar disorder (BD), major depressive disorder (MDD), and schizophrenia (SCZ) affect >1%, 6%, and ≈1% of the population, respectively (Malhi and Mann, 2018; Vieta et al., 2018; Marder and Cannon, 2019). Differentiation between these three major psychiatric diseases disorders is typically based on symptoms and course patterns. However, overlapping foundations, heterogeneous clinical features, and common underlying genetic vulnerabilities challenge traditional definitions of related nosological boundaries (Cross-Disorder Group of the Psychiatric Genomics, 2013; Cheng et al., 2018). The pleiotropic nature and the influence of gene-environment interactions on pathophysiology has focused attention towards a more comprehensive understanding of psychiatric illness and a pharmacogenomics approach of existing treatment strategies (Lopez-Leon et al., 2008; Serretti and Mandelli, 2008; Gatt et al., 2015; Harrison, 2015).

**Figure 1:**
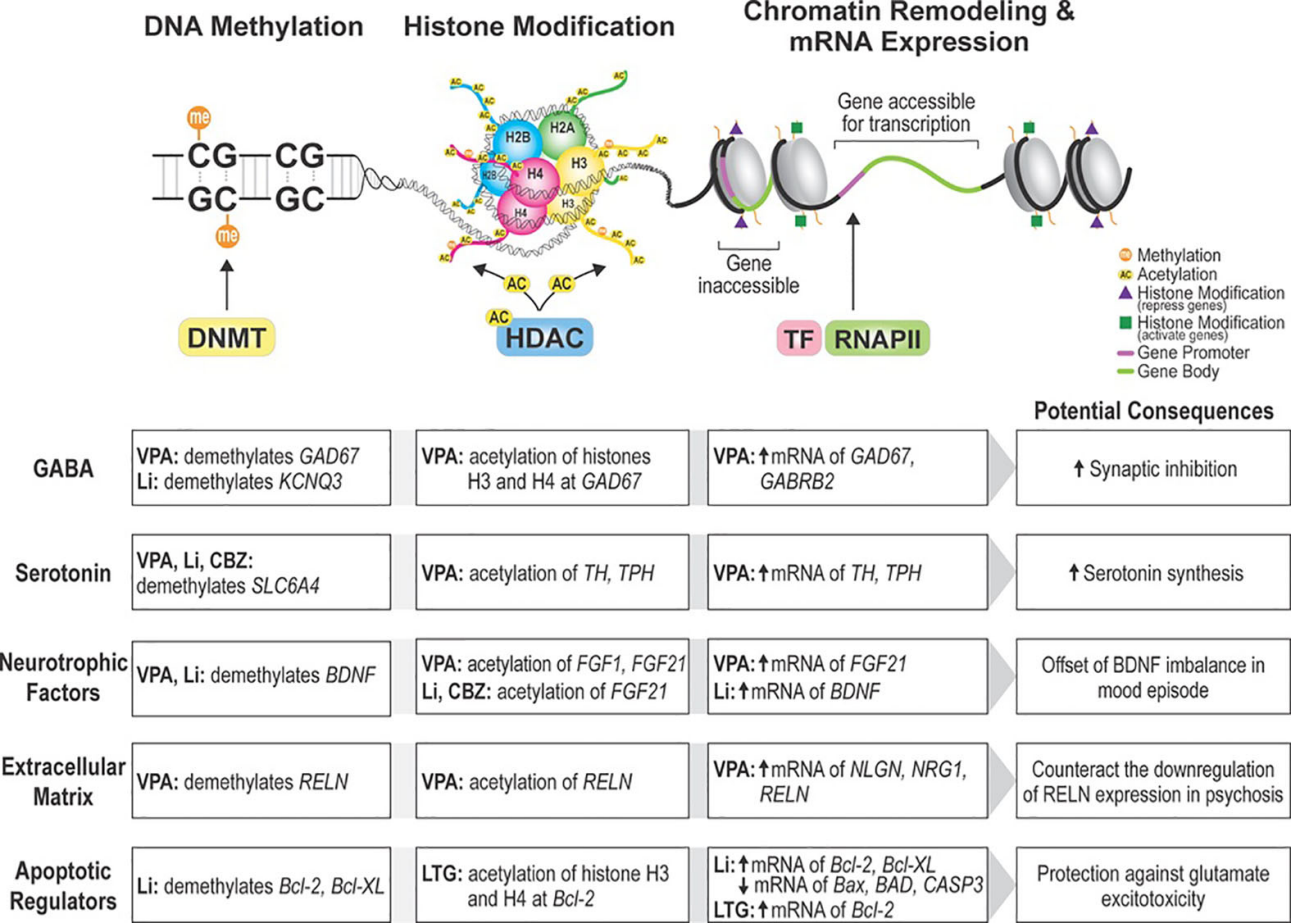
Pathways epigenetically impacted by mood stabilizers. Non-antipsychotic mood stabilizers alter the epigenetic expression of a variety of candidate genes in bipolar disorder and schizophrenia. Findings from our systematic review suggest that epigenetic changes induced by mood stabilizers produce neuroprotective effects through different pathways. DNMT, DNA methyltransferase; HDAC, histone deacetylase; TF, transcription factor; RNAPII, RNA polymerase II; VPA, valproic acid; Li, lithium; CBZ, carbamazepine; LTG, lamotrigine; GAD67, glutamate decarboxylase 67; KCNQ3, potassium voltage-gated channel subfamily Q member 3; GABRB2, gamma-aminobutyric acid receptor subunit beta-2; SLC6A4, sodium-dependent serotonin transporter and solute carrier family 6 member 4; TH, tyrosine hydroxylase; TPH, tryptophan hydroxylase; FGF21, fibroblast growth factor 21; BDNF, brain-derived neurotrophic factor; RELN, reelin; NLGN, neuroligin; NRG1, neuregulin 1; Bcl-2, Bcl-2 apoptotic regulator Bcl-XL- BCL2 Like 1; Bax, BCL2 associated X, apoptosis regulator; BAD, BCL2 associated agonist of cell death; CASP3, caspase 3.

Epigenetic changes involve reversible chromatin rearrangements that induce mitotically heritable, stable, long-term, and reversible gene expression patterns without altering the DNA sequence (**Figure 1**) (Bernstein et al., 2010; Allis and Jenuwein, 2016). DNA methylation and histone modifications are the most studied epigenetic marks in physiological and pathological contexts (Schenkel et al., 2017). In DNA methylation, DNA methyltransferases (DNMT) transfer a methyl group to 5′-cytosine residues at cytosine-guanine sequences (CpG) which are clustered in CpG islands, often unmethylated and located within gene promoters of active transcription and tumor suppressor genes (Inbar-Feigenberg et al., 2013; Houtepen et al., 2016). Acetylation and deacetylation of histones through histone deacetylases (HDACs) and histone acetyltransferases (HATs) activity regulate chromatin structure and gene expression (Eyal et al., 2004; Machado-Vieira et al., 2011; Inbar-Feigenberg et al., 2013; Schenkel et al., 2017). Whilst increased histone acetylation and decreased DNA methylation are associated with active transcription and gene expression, histone deacetylation and DNA hypermethylation are indicators of heterochromatin (condensed state of chromatin) and gene silencing (Machado-Vieira et al., 2011; Inbar-Feigenberg et al., 2013). Similarly, some studies on epigenetic modifications are based on the hypotheses that abnormal RNA expression is linked with altered epigenetics at gene promoter regions and regulatory sequences (Houston et al., 2013).

In physiological circumstances, epigenetic mechanisms control neurobiological processes but deregulation in these mechanisms can translate into an increased risk of disease development (Bernstein et al., 2010; Davies et al., 2012). Epimutations secondary to gene-environment interactions have been described to have a key role in the pathophysiology of major psychiatric disorders (Abdolmaleky et al., 2019) where aberrant DNA methylation and histone modification patterns have been identified. Histone acetylation levels are significantly altered in individuals with BD and, to a lesser extent, in individuals with SCZ (Gavin and Sharma, 2010; Machado-Vieira et al., 2011).There is also a reduction in global and specific DNA methylation levels in individuals with BD, MDD, and SCZ (Alladi et al., 2018). *In vitro* and *in vivo* studies have suggested the potential of mood stabilizers to reverse epimutations in major psychiatric disorders (Pisanu et al., 2018) making them a target for further research.

Classic mood stabilizers comprising of lithium, valproate, lamotrigine, and carbamazepine, which show antimanic, antidepressant and prophylactic effects, have been characterized as the mainstay of treatment for BD and as aides in MDD and SCZ (Bauer and Mitchner, 2004; Goodwin and Malhi, 2007). While mechanisms of action of valproic acid (VPA), carbamazepine (CBZ), lamotrigine (LTG), and lithium (Li) are not completely understood, there is robust evidence on their ability to target altered epigenetic functions (Seo et al., 2014; Houtepen et al., 2016; Pisanu et al., 2018) involved in the pathophysiology of BD, MDD and SCZ (Higuchi et al., 2011; Ludwig and Dwivedi, 2016). The putative neuroprotective and neurotrophic actions of Li are thought to be induced through epigenetic mechanisms that enhance the expression of molecules involved in neuroplasticity and cytoprotective proteins (Chuang et al., 2002; Schloesser et al., 2012). Likewise, identification of VPA as a class I and IIa HDAC inhibitor (Gottlicher et al., 2001; Phiel et al., 2001) suggests that associated reversion of HDAC-dependent transcriptional repression and histone hyperacetylation could be involved in its mood-stabilizing properties (Gavin and Sharma, 2010; Machado-Vieira et al., 2011). Less studied are the mechanisms of action of LTG and CBZ; neuroprotective effects of LTG exerted through upregulation of excitatory amino acid transporter activity (Schloesser et al., 2012; Leng et al., 2013) and increased global DNA methylation induced by CBZ (Pisanu et al., 2018) are the best described epigenetic changes. The histone deacetylase inhibitory properties of anticonvulsants (Eyal et al., 2004) and the potent antioxidant effects of lithium (Leng et al., 2008; Dwivedi and Zhang, 2015) have been postulated as potential pathways to reverse dysfunctional epigenetic regulation and variability in treatment response (Machado-Vieira et al., 2011).

### Objectives and Research Question

Studies on the epigenetic impact on candidate genes of mood stabilizers, especially Li and VPA, have consistently increased in the past decade but attempts to summarize the findings have been scarce (Alladi et al., 2018; Pisanu et al., 2018). This systematic review provides a qualitative summary of the current state of knowledge of the epigenetic effects of non-antipsychotic mood stabilizers in MDD, BD, and SCZ in an attempt to define the specific mechanisms through which these agents act at the epigenomic level.

## Methods

### Study Design

We developed the systematic review protocol based on the Preferred Reporting Items for Systematic Review and Meta-Analysis Protocols (PRISMA-P) 2015 (Shamseer et al., 2015) and conducted a comprehensive literature search of PubMed and EMBASE from their inception through 30 September 2019.

### Search Strategy

The following search string was used: (“epigenetic” OR “epigenomic” OR “DNA methylation” OR “DNA hydroxymethylation” OR “histone acetylation” OR “histone deacetylation” OR “histone methylation”) AND (“lithium” OR “carbamazepine” OR “lamotrigine” OR “mood stabilizer” OR “valproic acid”) NOT “cancer”. Search strategy for valproic acid was narrowed using the Boolean operator NOT, to exclude studies related to use of VPA as an epigenetic cancer drug.

**Figure 2:**
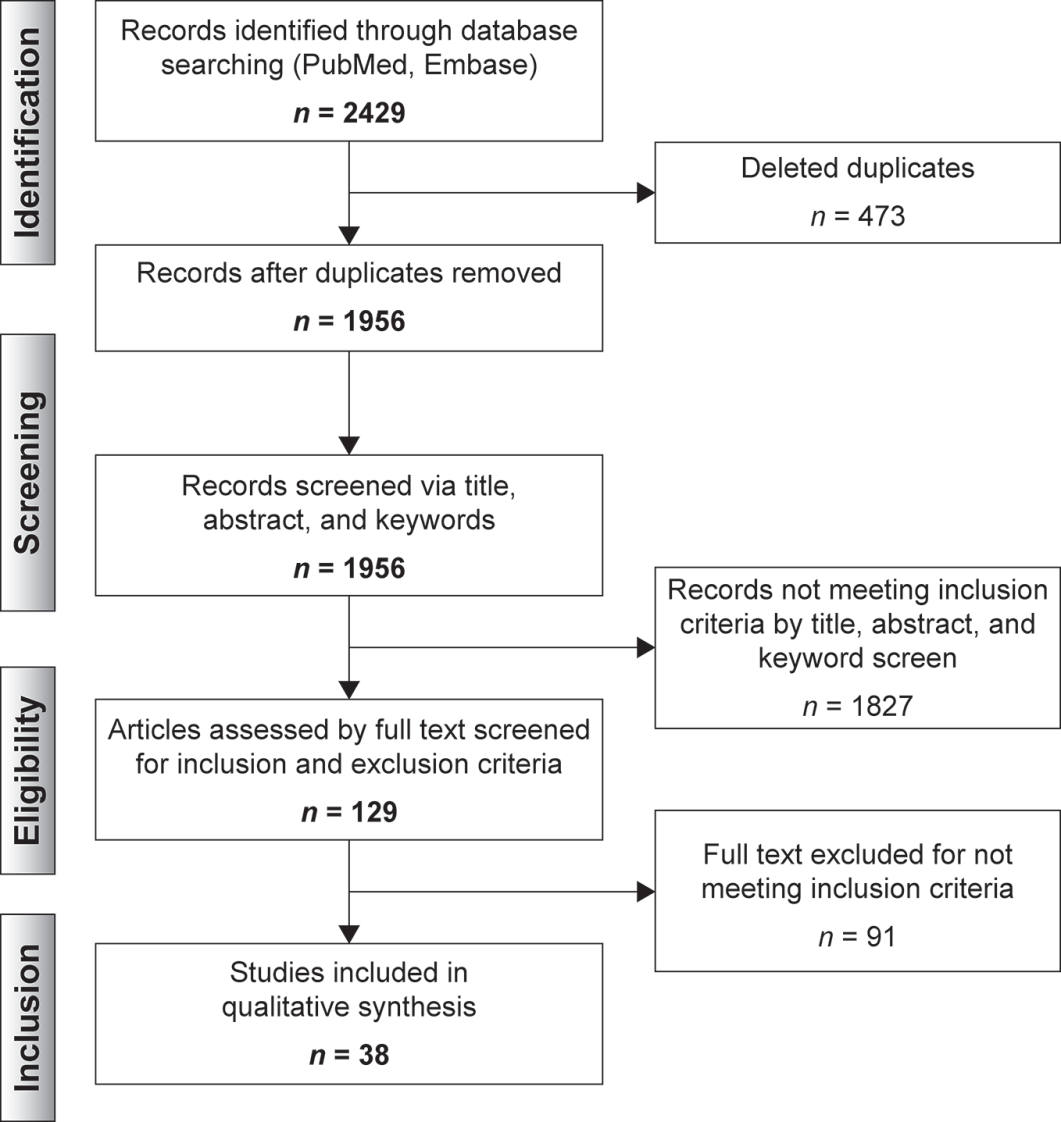
Flowchart based on PRISMA.

Articles were collated in Rayaan QCRI (Ouzzani et al., 2016). Duplicates were eliminated by the software. Each abstract was reviewed, through a blinded process, for eligibility by two independent reviewers (M.G.R. and M.U.K.). Disagreements were resolved *via* discussion and a senior reviewer (M. V.) assessing the relevance of the selected data. A total of 129 articles were selected for full-text review (**Figure 2**).

### Participants, Interventions, Comparators

Literature eligible for inclusion were (a) published articles with original data in English; (b) about gene-specific studies with patients, human-tissue, or mammalian models involving assessment of epigenetic changes with use of lithium, divalproex, carbamazepine, or lamotrigine for major psychiatric disorders (psychotic disorders, bipolar disorder, and depression) that (c) met a minimum quality standard of moderate or higher. The three selected major psychiatric disorders were chosen due to phenotypic similarities and shared symptomatology. Exclusion criteria were (a) reviews or non-experimental papers, (b) papers that did not report specific epigenetic changes, (c) papers not focused on the aforementioned psychiatric disorders, or (d) literature only published as abstracts or conference summaries.

### Data Sources, Studies Sections and Data Extraction

Each paper was reviewed for the nature of research, the species and tissue examined, sample size, mood stabilizer, targeted gene, epigenetic changes found, and associated psychiatric disorder.

## Results

### Study Selection and Characteristics

Thirty-eight articles met all the inclusion criteria; studies retrieved for the review were organized in a flow diagram (**Figure 2**). Twenty-one described studies of rodent samples (Tremolizzo et al., 2002; Dong et al., 2005; Tremolizzo et al., 2005; Kim et al., 2008; Dong et al., 2010; Hobara et al., 2010; Perisic et al., 2010; Matrisciano et al., 2011; Wang et al., 2012; Zimmermann et al., 2012; Calabrese et al., 2013; Leng et al., 2013; Ookubo et al., 2013; Liu et al., 2014; Mackowiak et al., 2014; Balasubramanian et al., 2015; Bator et al., 2015; Dwivedi and Zhang, 2015; Lee et al., 2015; Leng et al., 2016; Bahna and Niles, 2017). Seven articles studied human cell lines (Asai et al., 2013; Kao et al., 2013; Dyrvig et al., 2017; Zong et al., 2017; Billingsley et al., 2018; Dyrvig et al., 2019; Manca et al., 2019) and eight focused on blood samples of subjects diagnosed with either of the selected psychiatric disorders (Gavin et al., 2009; D’Addario et al., 2012; Dell’Osso et al., 2014; Huzayyin et al., 2014; Burghardt et al., 2015; Burghardt et al., 2016; Houtepen et al., 2016; Bengesser et al., 2018). One article studied rodents and human cell lines and subjects (Kaminsky et al., 2015) and one focused on patients and human cell lines (Kakiuchi et al., 2003). The papers are summarized in **Tables 1**–**4**.

**Table 1:** Summaries of animal studies included in the systematic analysis.

Reference(by year)	Nature of research	Species, tissue	Mood stabilizer	Targeted gene	Data extracted
(Bahna and Niles, 2017)	Examine mechanisms underlying the upregulation of melatonin MT1 receptors by VPA.	Rat, glioma cells	VPA	*MTNR1A*	VPA induces an upregulation of the MT1 receptor through a mechanism involving histone H3 acetylation on the *MT1* promoter.
(Leng et al., 2016)	To investigate the association between *FGF21* expression and mood stabilizer’s histone deacetylase inhibition in glial cells as well as to identify the HDAC isoform(s) involved in the process.	Rat, glioma cells and cortical glial cells	VPA, Li, LTG, CBZ	*FGF21*	In C6 cells VPA significantly increased (up to 35-fold) levels of *FGF21* mRNA in a dose- and time-related manner. Li induced a weak (2- to 3-fold) increase in *FGF21* mRNA levels at high concentrations. LTG and CBZ were ineffective. VPA and, less significantly, LTG increased acetylation of histone 3 levels. CBZ produced a modest dose-dependent increase, while Li did not produce significant changes. In primary glia VPA dose-dependently increased *FGF21* mRNA levels.
(Bator et al., 2015)	Explore the effect of VPA on histone acetylation in a neurodevelopmental animal model of schizophrenia.	Rat; adult medial prefrontal cortex tissue	VPA	H3K9ac, HDAC2	VPA administration did not affect the decrease in H3K9ac nor acetylation level of H3K9 but prevented the MAM induced increase in HDAC2 immunoreactivity at P70.
(Balasubramanian et al., 2015)	Evaluate the potential epigenetic effects of mood stabilizers on the expression of the BH4 pathway gene *Spr*	Rat, serotoninergic cell line (RN46A)	VPA, LTG, CBZ, and Li	*Spr*	VPA and Li increased *Spr* mRNA expression. VPA also increased intracellular BH4 levels and acetylation at K3K9/K14ac histone mark in the *Spr* promoter region. CBZ and LTG did not result in any significant change in mRNA expression.
(Lee et al., 2015)	To identify genes affected by Li and VPA and assess the epigenetic mechanisms involved in their mechanism of action. Determine whether chronic exposure to Li and VPA could induce histone modifications in the *Lepr* promoter.	Rat, hippocampal tissue	VPA, Li	*Lepr*	Increased *Lepr* expression with Li (32.6%) and VPA (127.4%). Both drugs produced histone H3 methylation and acetylation in *Lepr*.
(Dwivedi and Zhang, 2015)	Aimed to delineate Li’s epigenetic impact on the expression of *BDNF* gene and other pro and anti-apoptotic genes.	Rat, hippocampal neurons	Li	*BDNF, BAX, Bcl-2, Bcl-XI, BAD, CASP-3*	Li produced a dose-dependent increase in mRNA expression of *BDNF* and exon IV, while methylation was decreased in exon IV. Expression of anti-apoptotic genes *Bcl-2* and *Bcl-Xl* was increased while expression of pro-apoptotic genes *Bax*, *BAD*, and *caspase 3* was decreased with Li exposure.
(Liu et al., 2014)	Investigate the hypothetical interrelation between histone acetylation modification and expression of *TH* and *TPH* gene in CUS-induced depression in rats.	Rat, hippocampal tissue	VPA	*TH, TPH*	VPA prevented a decrease of H3 and H4 acetylation and an increase of HDAC5 protein expression in CUS rats. VPA clearly inhibited decrease of *TH* protein and mRNA expression but only partly reversed the decrease of *TPH* protein and mRNA expression.
(Mackowiak et al., 2014)	Investigate the epigenetic processes in a neurodevelopmental model of SCZ based on the gestational administration of MAM. An additional pharmacological study was performed to determine the period in adolescence critical for developing dysfunction in histone H3 methylation in the adult offspring.	Rat, medial prefrontal cortex	VPA	H3K4me3, *ASH2L*, *JARID1c*	VPA decreased *JARID1c* protein levels. Early exposure to VPA prevented the expected decrease in H3K4me3 and *ASH2L* protein induced by MAM but did not affect the *JARID1c* levels.
(Leng et al., 2013)	To investigate the neuroprotective effects of LTG exerted *via* chromatin remodeling through HDAC inhibition and up-regulation of *Bcl-2*.	Rat, cerebellar granule cells	LTG	Histones H3 and H4, *Bcl-2*	LTG produced a time-dependent increase in the acetylation levels of histones H3 and induced a moderate decrease in HDAC activity. LTG induced a dose-dependent increment of *Bcl-2* mRNA and protein levels.
(Ookubo et al., 2013)	Determine similar region-specific effects on tissue monoamine concentrations or protein expression of AcH3 and HDACs with antidepressants and mood stabilizers and identify the relation of HDAC and specific antidepressant-like effects in brain regions.	Mouse, brain	VPA, Li, CBZ and LTG	AcH3, HDACs	AcH3 protein expression was significantly increased with VPA, Li, and LTG in cingulate cortex and nucleus accumbens; no effect was observed with CBZ and LTG in amygdala. In striatum, expression of HDAC-2, -3, and -8 was increased with CBZ and LTG while Li induced decreases of HDAC-1, -3, -4, -5, -7, -8, and -10. HDAC-2 and -3 were increased in nucleus accumbens after exposure to CBZ and LTG. In hippocampus, VPA, Li, and LTG decreased expression of HDAC-5 and -7. In cingulate, VPA, CBZ, and LTG increased HDAC-1, HDAC-3, and HDAC-5 protein expression. The former two were also increased by CBZ and LTG in amygdala.
(Calabrese et al., 2013)	Explore the neuroadaptive changes produced by chronic single or combinatory therapy with lurasidone and VPA.	Rat, ventral, and dorsal hippocampal tissue	VPA alone or in combination with lurasidone	*BDNF*, *Arc*, and HDAC-1, -2 and -5	In ventral hippocampus, VPA increased the long 3′ UTR *BDNF* mRNA levels and more robustly in combination with lurasidone. Expression levels of exon IV was increased with VPA alone and in combination but mRNA levels of exon IV were reduced with VPA alone. In dorsal hippocampus, VPA, alone and in combination, increased total *BDNF* mRNA levels. VPA significantly increased proBDNF and mature BDNF levels in this sub region. VPA alone and in combination upregulated mRNA levels of *Arc* in hippocampus. The gene expression of HDACs, mainly HDAC-2 and -5, was modified with the combinatory therapy.
(Wang et al., 2012)	Investigate the effect of VPA on the mRNA levels of two excitatory post-synaptic cell adhesion molecules and two extracellular matrices in primary astrocyte cultures.	Rat, neuronal, astroglial, and neuro-glial mixed culture systems	VPA	*NLGN*, *NRG1*, *TSP-3*, and *NPTX1*	VPA increased levels of *NLGN*, *NRG1*, *NPTX1*, and *TSP-3* mRNA in a time- and concentration-dependent manner in astrocytes.
(Zimmermann et al., 2012)	Explore the inhibitory effects of antidepressants, VPA and CBZ on DNMT activity in primary astrocytes from rat cortex.	Rat, primary astrocytes	VPA and CBZ	DNMT	Neither VPA nor CBZ decreased DNMT activity in astrocytes.
(Matrisciano et al., 2011)	Explore the pharmacological activation of mGlu2/3 receptors on the epigenetic regulation of genes linked to the pathophysiology of schizophrenia.	Mouse, brain	VPA	*Gadd45-B*	Exposure to VPA produced a 2- to 3-fold increase of *Gadd45-B* mRNA brain levels. VPA increased the binding of *Gadd45-B* to reelin, *GAD67* and *BDNF-IX* promoters.
(Hobara et al., 2010)	To investigate the effect of antidepressants and mood stabilizers on the mRNA levels of HDACs in mouse leukocytes.	Mouse, leukocytes	Li and VPA	HDAC-2, -4, -5, -6, and -8 mRNA	The expression of HDAC2. HDAC4, HDAC5, HDAC6, and HDAC8 mRNA of mice receiving either Li or VPA were comparable to those of control mice.
(Dong et al., 2010)	To explore if the demethylation of RELN and Gad67 promoters induced by VPA is the result of induction of DNA demethylation mechanisms or reduced DNMT activity.	Mouse, frontal cortex	VPA	*GAD67* and *RELN*, DNMT1 and DNMT3a	VPA failed to induce significant changes in DNMT1 and DNMT3a mRNA levels but produced DNA demethylation activity as well as an upregulation of *RELN* and *GAD67* mRNA expression.
(Perisic et al., 2010)	Analyze the potential of mood stabilizers to affect epigenetic parameters in astrocytes by measuring global histone H3 and H4 acetylation/methylation, DNA methylation and *GLT-1* promoter.	Rat, primary astrocytes from hippocampus and cortex	VPA, CBZ, and LTG	Global histone acetylation and DNA methylation, *GLT-1*	Only VPA induced global histone H3 and H4 transient hyperacetylation and significant demethylation. LTG did not produce changes of DNA methylation. VPA did not change DNMT-1 levels in astrocytes and slightly reduced levels of dimethyl-H3K9. Exposure to CBZ did not inhibited class I or class II HDACs. VPA increased GLT-1 mRNA in a dose dependent manner, while LTG did not influence *GLT-1* mRNA expression.
(Kim et al., 2008)	Examine expression of melatonin MT1 receptor and selected epigenetic modulators after exposure to clinically relevant concentrations of VPA.	Rat, glioma cells	VPA	*MT1* receptor, *MeCP2*, HDAC-1, -2, and -3	Significant time-dependent increases in mRNA expression after exposure to VPA was reported in melatonin *MT1* receptor, *MeCP2*, HDAC1, 2, and 3.
(Tremolizzo et al., 2005)	Study if VPA-induced hyperacetylation of chromatin histone tails can prevent hypermethylation of reelin promoter and schizophrenia-like behavioral traits induced by methionine in mice.	Mouse, frontal cortex	VPA	*RELN*	VPA induced an increase of acetylated H3 in frontal cortex and prevented H3 hypermethylation. VPA reversed the methionine-induced hypermethylation from the reelin promoter region and prevented reelin mRNA downregulation.
(Dong et al., 2005)	To test if pretreatment with VPA revert the downregulation of *RELN* and *GAD67* expression induced by MET and the accompanying hypermethylation of these genes.	Mouse, frontal cortex	VPA	*GAD67* and *RELN*	VPA increased levels of acetylated-H3 flanking *RELN* and *GAD67* promoter sites and upregulated the expression of these genes by decreasing DNA methylation-dependent chromatin remodeling.
(Tremolizzo et al., 2002)	Explore whether administration of VPA may revert the decrease in *RELN* and *GAD67* expression secondary to methionine administration in mice.	Mouse, frontal cortex	VPA	*RELN*, *GAD67*, *MeCP2*	VPA reverted L-methionine-induced down-regulation of *RELN* and *GAD67* in both mouse samples and increased acetylation of histone H3.

M1, Metallothionein-1M; VPA, valproic acid; Li, lithium; FGF21, fibroblast growth factor 21; LTG, lamotrigine; CBZ, carbamazepine; MAM, methylazoxymethanol acetate; DNMT, DNA methyltransferases; HDACs, histone deacetylases.

**Table 2:** Summaries of studies in human cell lines included in the systematic analysis.

Reference (by year)	Nature of research	Species, tissue	Mood stabilizer	Targeted gene	Data extracted
(Dyrvig et al., 2019)	Explore the epigenetic regulation of *CHRNA7* as a response predictor and modulator to a7 nAChR agonists.	Human, adenocarcinoma and neuroblastoma cells	VPA	*CHRNA7*	VPA caused transcriptional upregulation of *CHRNA7*, which correlated with decreased DNA methylation. Concomitant administration of VPA and nicotine in SH-SY5Y cells increased *CHRNA7* expression and decreased methylation levels.
(Manca et al., 2019)	Analyze *MAOA* regulation in a human female heterozygous cell line to explore the transcriptional and epigenetic variation at the uVNTR domain in *MAOA* in response to sodium VPA.	Human, neuroblastoma cells	VPA	uVNTR *MAOA*	Altered methylation pattern at the uVNTR domain when exposed to sodium VPA.
(Billingsley et al., 2018)	Cells were exposed to cocaine or Li to determine if transcriptional activity at *CACNA1C* locus was regulated in a stimulus-inducible manner.	Human, neuroblastoma cells	Li	*CACNA1C*	Exposure to Li increased expression from all three *CACNA1C* promoter gene constructs, which encode for Cav1.2.
(Zong et al., 2017)	Assess response of *GABRB2* mRNA expression alterations to epigenetic modifications with 5-azacytidine or VPA.	Human, neuroblastoma cells	VPA	*GABRB2*	VPA caused an upregulation of *GABRB2* mRNA expression accompanied by histone 4 hyperacetylation at the *GABRB2* Yi6 region.
(Dyrvig et al., 2017)	Explore the effect of three different mood stabilizers on *BRD1* expression and changes in DNA methylation.	Human, neuroblastoma cells	VPA, Li, and CBZ	*BRD1*	Li caused a 13% decreased expression of *BRD1* exon 1B containing transcripts. VPA caused a 10% increase of the expression of *BRD1* exon 1A. CBZ caused a 15% increase in expression of total *BRD1*.
(Asai et al., 2013)	Perform a comprehensive and site-specific analysis (DNA methylation status) of the epigenetic effects of three mood stabilizers.	Human, neuroblastoma cells	VPA, Li, and CBZ	*BDNF* and *SLC6A4*	Comprehensive analysis indicated that all three mood stabilizers had a propensity to increase DNA methylation. All three stabilizers were associated with hypomethylation of *SLC6A4* CpG3 and CpG4, while level of DNA methylation of promoter IV of *BDNF* was not significantly affected by any of the studied drugs.
(Kao et al., 2013)	To investigate the activation of *FGF1* 1B promoter by VPA through inhibition of HDAC and GSK-3 activities.	Human, glioblastoma cells	VPA and Li	*FGF1*, *RFX2*, and *RFX3*	VPA-treated cells showed an increased expression of *RFX2* and *RFX3*, which significantly increased *FGF1* 1B expression. VPA activated *FGF1* 1B promoter activity through inducing histone acetylation around the RR2 regulatory region. Li enhanced the expression of F*GF1* 1B and *RFX2* though inhibition of GSK-3 activity.

VPA, valproic acid; CBZ, carbamazepine; Li, lithium.

**Table 3:** Summaries of studies on human subjects affected by a major psychiatric disorder included in the systematic analysis.

Reference (by year)	Nature of research	Species, tissue	Mood stabilizer	Targeted gene	Data extracted
(Bengesser et al., 2018)	Evaluate and compare methylation of *ARNTL* between bipolar disorder and controls.	Human, blood	Li	*ARNTL*	Significant association between methylation *ARNTL* region cg05733463 and Li intake.
(Houtepen et al., 2016)	To examine the DNA methylation signatures of psychotropic drugs in BD patients through genome-wide and candidate-genes approaches.	Human, PBMC	Li, LTG, VPA, CBZ	*RELN*, *SLC1A2*, *MTNR1A*, *IGF2*, *H19*, *BDNF*, *SLC6A4*, and *GAD1*.	Association between specific drugs and loci methylation status did not provide any replication for the candidate genes after false discovery rate correction, most likely as a consequence of limited power.
(Burghardt et al., 2016)	Identify and validate a candidate gene associated with atypical antipsychotics-induced insulin resistance through a cross sectional approach of subjects with BDI treated with Li monotherapy or atypical antipsychotics.	Human, peripheral blood	Li	*FAR2*, global DNA methylation	No association between *FAR2* methylation levels and an insulin-resistant state in subjects treated with Li, which may be affected by the small sample size in this group.
(Burghardt et al., 2015)	To examine the relationship between atypical antipsychotic or mood stabilizer therapy and insulin resistance and degree of peripheral blood DNA methylation in subjects with BDI.	Human, leukocytes	LTG, Li, VPA	Global DNA methylation	Regarding the mood stabilizer group, global methylation values were not significantly reduced, which may be affected by the small sample size in this group.
(Dell’Osso et al., 2014)	Cross-sectional analysis of differences in *BDNF* promoter gene methylation in patients with mood disorders. DNA methylation was also analyzed on the basis of the pharmacotherapy.	Human, PBMC	Li, VPA	*BDNF*	Both Li and VPA showed a not significant decrease in DNA methylation level at *BDNF* gene promoter when compared to antidepressants and atypical antipsychotics. Lower methylation levels seen in BD I subjects could be associated with the fact that they were mainly treated with mood stabilizers. Relation between mood stabilizer dose and methylation level was not specified.
(Huzayyin et al., 2014)	Investigate the relationship between DNA methylation in patients with BD and excellent response to Li, their affected and unaffected relatives and controls.	Human, lymphoblast	Li	Global DNA methylation, *GPx*	Global methylation was decreased in BD subjects and their relatives compared to control subjects.
(D’Addario et al., 2012)	Investigate role of DNA methylation in the regulation of *BDNF* transcription and assess differences across pharmacological treatment and other groups.	Human, PBMC	Li, VPA	*BDNF*	DNA methylation was significantly reduced in subjects under therapy with Li or VPA compared with treatment with other drugs. *BDNF* mRNA levels were not measured.
(Gavin et al., 2009)	Investigate an *in vitro* and an *in vivo* approach for measuring chromatin remodeling in real clinical time. In vitro approach was performed through cultured human lymphocytes with VPA and the *in vivo* approach studied SCZ and BD subjects treated with VPA for 4 weeks.	Human, lymphocytes	VPA	*GAD67*, H3K9,K14ac	In vitro exposure to VPA significantly increased H3K9,K14ac protein levels and *GAD67* expression. VPA treatment for 4 weeks significantly increased H3K9,K14ac protein levels across all subjects, being more notorious in BD subjects. VPA showed a dose response effect on the mRNA expression of *GAD67* and H3K9,14ac protein levels.

VPA, valproic acid; LTG, lamotrigine; CBZ, carbamazepine; Li, lithium.

**Table 4:** Summaries of studies on more than one type of populations included in the systematic analysis.

Reference (by year)	Nature of research	Species, tissue	Mood stabilizer	Targeted gene	Data extracted
(Kaminsky et al., 2015)	Explore the association of mood stabilizers with epigenetic changes in *KCNQ3* gene.	Human, postmortem PFC tissue Human, neuroblastoma cells Rat, PFC tissue	VPA, Li	*KCNQ3*	In human sample, Li or VPA increased DNA methylation. Treatment of neuroblastoma cells did not produced significant methylation changes with either stabilizer. In rats, Li was associated with a small significant elevation of mean *KCNQ3* exon 11 DNA methylation.
(Kakiuchi et al., 2003)	To determine the contribution of *XBP1* gene and *ATF6*, the gene upstream of *XBP1*, as risk factors for BD and the effect of mood stabilizers on gene expression.	Human, lymphoblasts and neuroblastoma cells	VPA, Li, and CBZ	*ATF6* and *HSPA5*	Of the three mood stabilizers, only VPA had a significant effect on *ATF6* mRNA expression but not *HSPA5* mRNA levels.

VPA, valproic acid; CBZ, carbamazepine; Li, lithium.

### Synthesized Findings

#### Rodent Models

SCZ-like epigenetic modifications and symptoms, namely positive, negative, and cognitive, have been replicated in animal samples through the administration of L-methionine (MET) and methylazoxymethanol acetate (MAM) to obtain models for SCZ. Both VPA and antipsychotics have shown a potential reversibility effect of symptoms and epigenetic markers on MET or MAM treated rodents (Tremolizzo et al., 2002; Lodge, 2013; Wang et al., 2015).

Five papers examined the impact of mood stabilizers on genes involved in the regulation of histone activity (Kim et al., 2008; Hobara et al., 2010; Ookubo et al., 2013; Mackowiak et al., 2014; Bator et al., 2015). Kim et al. (Kim et al., 2008) exposed rat C6 glioma cells to prolonged treatment with low clinically relevant doses of VPA (0.5 mM or 1 mM), reporting significant increases in HDAC-1, -2, and -3 mRNA expression (class I HDAC isoforms), as well as increases in methyl-CpG binding protein 2 (*MeCP2)* and Metallothionein-1M (MT_1_) mRNA expression. These findings suggest a compensatory temporal upregulation following the HDAC inhibitory effects of VPA as well as a relation between HDACs involvement in the melatonin MT_1_ receptor induction. Similarly, Hobara et al. (2010) investigated the effect of Li and VPA in class I and II HDACs (HDAC-2, -4, -5, -6, and -8) in lymphocytes from male C57BL/6 and BALB/c mice exposed to a forced swim test, a highly reliable model of depressive-like behavior (Yankelevitch-Yahav et al., 2015). After 21 days of treatment, expression of HDAC-2, -4, -5, -6, and -8 mRNA levels were comparable to those of stress-free control mice, suggesting a non-selective nature of Li and VPA as HDAC inhibitors, and that they do not have a causal role in the altered expression of HDACs in mood disorder patients (Hobara et al., 2010). The region-specific changes in histone deacetylase expression and histone H3 acetylation (AcH3) induced by VPA, Li, CBZ, and LTG in C57BL/6 mice was investigated in another study (Ookubo et al., 2013). Treatment with VPA, Li, and LTG significantly increased AcH3 expression in nucleus accumbens and cingulate cortex, while CBZ and LTG increased HDAC-2 and -3 in striatum; VPA, CBZ, and LTG increased HDAC-3 in cingulate cortex and HDAC-5 in the amygdala (Ookubo et al., 2013). The latter shows epigenetic changes associated with modified HDAC expression and that transcriptional activation includes an alternating recruitment of histone acetyltransferases and HDACs, rather than a process of increasing acetylation only (Shahbazian and Grunstein, 2007). Impact on histone H3 methylation in the prefrontal cortex was studied in MAM-injected Wistar male rats through measurements of *ASH2L* levels, which encodes for Set1/Ash2 histone methyltransferase complex subunit ASH2, and lysine-specific demethylase 5C enzyme, JARID1c (Mackowiak et al., 2014). Use of VPA in early adolescence prevented disturbances in *ASH2L* and H3K4me3 but did not affect the levels of *JARID1c*, a key protein in the demethylase activity on H3K4me3 (Mackowiak et al., 2014; Rondinelli et al., 2015). The same group reported that VPA prevented an increase in HDAC-2 levels evoked by MAM but not H3K9 acetylation levels (Bator et al., 2015). An additional paper studied the impact of CBZ and VPA on DNMT1 activity in primary astrocytes from rat cortex, reporting that neither of the agents altered the expression levels of DNMT (Zimmermann et al., 2012).

Chronic unpredictable stress (CUS)–induced depression in rats was used in a study of epigenetic histone modification and gene expression induced by VPA on tyrosine hydroxylase (*TH*) and tryptophan hydroxylase (*TPH*), enzymes involved in the biosynthesis of L-DOPA (a precursor to norepinephrine) and serotonin respectively (Liu et al., 2014). In controls, CUS led to decreased acetylation of H3K9 and H4K12, and reduced expression of *TH* and *TPH*. Administration of VPA reversed these downregulations and prevented an increase of HDAC-5 in the hippocampus. Regulation of another gene involved in the monoamine neurotransmitter formation, *Spr*, associated with increased susceptibility to mood disorders, was studied by evaluating the epigenetic changes after exposure to VPA, Li, LTG, and CBZ (Balasubramanian et al., 2015). Exposing RN46A cells (derived from embryonic rat medullary raphe nucleus) to VPA and Li showed increased *Spr* mRNA expression, protein levels, and histone acetylation in the *Spr* promoter. The increases in *Spr* resulted in augmented levels of tetrahydrobioptherin (BH4), a cofactor involved in the biosynthesis of diverse neurotransmitters. Conversely, treatment with LTG and CBZ did not result in significant changes in *Spr* mRNA expression (Balasubramanian et al., 2015). A fourth study analyzed the potential of mood stabilizers VPA’s, CBZ’s, and LTG’s affect on global DNA methylation, histone acetylation, and methylation of the *GLT-1* promoter, which encodes for the excitatory amino acid transporter 2 (EAAT2), a research target for MDD and BD, in astroglial cultures of Sprague-Dawley rats (Perisic et al., 2010; Blacker et al., 2019). Only VPA induced histone hyperacetylation of H3 and H4, and DNA methylation, both reversible after drug removal. VPA also led to reduced *GLT-1*promoter methylation at three CpG sites, enriched acetylation of H3 and H4, and a dose dependent increase of *GLT-1* mRNA. LTG and CBZ failed to produce significant changes (Perisic et al., 2010).

A preliminary study by Wang et al. (2012) examined rat astrocytes and the effect of VPA on the synaptic excitatory/inhibitory balance through modulation of cell adhesion molecules (CAM) and extracellular matrices (ECM), which are known to be involved in the formation and maturation of synapses (Dalva et al., 2007). This paper evaluated VPA-induced changes on mRNA levels of neuroligin-1 (*NLGN*) and neuregulin-1 (*NRG1*), which are CAMs involved in the regulation of glutamatergic and GABAergic synapses (Craig and Kang, 2007), and neuronal pentraxin-1 (*NPTX1*) and thrombospondin-3 (*TSP3*), which are ECMs that promote synaptogenesis (Christopherson et al., 2005; Koch and Ullian, 2010). Exposure to VPA significantly increased the mRNA levels of the four molecules in a time- and concentration-dependent manner in astrocytes (Wang et al., 2012).

Activity on genes encoding for pro- and anti-apoptotic proteins have also been reported, highlighting them as a potential therapeutic target in BD, a disorder were neuronal resilience and plasticity cascades are altered (Schloesser et al., 2008). A novel study in Sprague Dawley rats identified that LTG had protective effects against glutamate excitotoxicity through HDAC inhibition and *Bcl-2* upregulation in primary neuronal cerebellar granule cells (CGC) (Leng et al., 2013). LTG pretreatment provided a dose- and time-dependent protection against glutamate toxicity and decrease in HDAC activity, sparing histone acetyltransferase (HAT) activity (Leng et al., 2013). LTG treatment caused moderate time-dependent increase of histones H3 and H4 acetylation levels. Inhibition of HDAC activity by LTG was less robust, however, than VPA-related inhibition. In this study, the anti-apoptotic *Bcl-2* gene was positively impacted with LTG treatment. Concentration-dependent increases in *Bcl-2* mRNA levels with an associated increase of Bcl-2 protein were observed after exposure of CGC cultures to incremental doses of LTG (0-100 µM) for 2 days (Leng et al., 2013).

The relevance of *Bcl-2* has risen since recent links between *Bcl-2* dysregulation and mood disorders were identified (Schloesser et al., 2008), and the mechanisms through which Li and VPA impact cellular plasticity cascades. One study described the influence of lithium on the expression of neuroprotective genes *BDNF*, *Bcl-2*, and *Bcl-XL* and pro-apoptotic genes *Bax*, *BAD* and *caspase-3* in rat hippocampal neurons (Dwivedi and Zhang, 2015). After exposure to Li, expression of *Bcl-2* and *Bcl-XL* genes were increased in a dose-dependent manner, while expression of *Bax*, *BAD*, and *caspase-3* was decreased by both doses of Li (1 mM and 2 mM), with magnitude of change being higher at 2 mM (Dwivedi and Zhang, 2015). The mRNA expression of *BDNF* was increased by 67% when exposed to Li at 1 mM and was fully sustained at 2 mM, especially on specific *BDNF* promoter exon IV, where methylation was decreased by Li; protein levels of *BDNF* were increased by 53% and 89% for 1 mM and 2 mM Li, respectively (Dwivedi and Zhang, 2015). One additional study focused on changes in *BDNF*, *Arc*, and epigenetic regulators HDAC-1, -2, and -5 in Sprague-Dawley rats’ hippocampi after exposure to VPA, lurasidone, or a combination of both (Calabrese et al., 2013). Regarding treatment with VPA only, an increase in *BDNF* mRNA levels in ventral and dorsal hippocampus was reported, as well as proBDNF and mature BDNF levels. *Arc* mRNA levels were also upregulated after exposure to VPA. *Arc* and *BDNF* expression increased more consistently with combination therapy, and HDAC isoform mRNA levels were significantly decreased (Calabrese et al., 2013).

Four papers examined the epigenetic changes secondary to VPA administration on *RELN* (reelin) and *GAD_67_* in MET-induced mouse models of SCZ (Tremolizzo et al., 2002; Dong et al., 2005; Tremolizzo et al., 2005; Dong et al., 2010). *RELN* expression, synthetized by GABAergic interneurons, is reduced in neocortex of SCZ and BD, paralleled by a down-regulation of *GAD_67_* levels, responsible for regulating cortical GABA levels (Guidotti et al., 2000; Guidotti et al., 2016). When comparing randomly sampled MET-injected populations of heterozygous *reeler* mice (reelin-deficient) versus wild type mice, VPA increased H3 histone acetylation, and reversed decreased mRNA expression of *RELN* and *GAD_67_* induced by L-methionine in prefrontal cortices of both populations. VPA failed to modify H4 acetylation and *GAD_65_* expression (Tremolizzo et al., 2002). Likewise, in Swiss albino mice’s frontal cortices, VPA prevented MET-induced *RELN* promoter hypermethylation and reduced the *MeCP2* binding to *RELN* and *GAD_67_* in frontal cortices (Dong et al., 2005). Upregulated *MeCP2* binding is associated with hypermethylation of CpG island promoters and increased recruitment of HDACs which favors gene transcriptional repression (Dong et al., 2005). VPA action on histone acetylation and methylation dynamics in *RELN* promoter was confirmed in MET-treated B6C3Fe male mice in which enhanced acetylation of H3 histone and prevention of MET-induced *RELN* hypermethylation and mRNA downregulation was observed after exposure to VPA (Tremolizzo et al., 2005). In this study, correction of L-methionine-related behavioral deficits was also observed after VPA treatment. More recently, the association between inhibition of *RELN* expression and DNA methyltransferases (DNMT) was explored in Swiss albino mice to better determine the pathway through which VPA induces demethylation of RELN and GAD_67_ (Dong et al., 2010). Although this study confirmed previous evidence of the association between overexpression of DNMT1 and DNMT3a in SCZ cortex and hypermethylation of the promoters of *RELN* and *GAD_67_ (*Veldic et al., 2004*)*, promoter demethylation induced by VPA was not caused by a decrease of DNMT activity or levels, but by increased acetylation of histones by HDAC inhibition (Dong et al., 2010). A fifth study examined the impact of VPA on growth arrest and DNA-damage-inducible beta (*Gadd45-β*) expression and binding properties (Matrisciano et al., 2011). In mice hippocampi, frontal cortices, and cerebellums, VPA was reported to robustly upregulate *Gadd45-β* expression by activation of DNA demethylation, a 2- to 3-fold increase of *Gadd45-β* mRNA levels, and an increased binding to reelin, *GAD_67_*, and *BDNF-IX* promoters (Matrisciano et al., 2011).

VPA- and Li-mediated epigenetic effects on *Lepr*, leptin receptor gene, implicated in regulation of mood and satiety, were explored in rats’ hippocampi (Lee et al., 2015). Both agents produced an augment in the expression of *Lepr*, although VPA showed a more significant increase (127.4% vs. 32.6%). Histone H3 methylation and acetylation were induced by VPA and Li; for Li, a more pronounced demethylation of H3 was observed. Another gene involved in metabolism, fibroblast growth factor 21 (*FGF21*), and its effect after exposure to VPA, CBZ, LTG, and Li was studied by Leng et al. (2016) in rat primary cortical glial cells and C6 glioma cells. C6 and glial cells treated with either VPA or Li experienced a dose-dependent increase in *FGF21* mRNA levels, while LTG and CBZ were ineffective. VPA, LTG and, less potently, CBZ significantly increased H3 acetylation levels (Leng et al., 2016). In another study, further examination of the mechanisms underlying melatonin *MT_1_* receptors’ upregulation by VPA was performed in rat glioma cells (Bahna and Niles, 2017). Exposure to VPA increased expression of *MT_1_* mRNA and acetylation of H3K9 across the receptor’s promoter.

#### In Vitro Human Cell Line Model

Study of human neuroblastoma and glioblastoma cells offer a window of what might happen *in vivo* and might be better models than HeLa adenocarcinoma cells to investigate the effects of mood stabilizers, due to their neuronal characteristics (Dyrvig et al., 2017).

*CHRNA7*, encoding the α7 nicotinic acetylcholine receptor (nAChR) has been linked to SCZ (Stephens et al., 2009). In one study, VPA was found to cause an upregulation of *CHRNA7* expression, correlating with decreased DNA methylation on HeLa cells, suggesting a potential reversing effect of pharmacotherapy on an epigenetic mechanism (Dyrvig et al., 2019). A dose-dependent effect was observed; while treatment with 0.3mM VPA produced a 4-fold upregulation of the expression, 0.6 mM VPA resulted in an 8.5-fold increase. Concomitant exposure to VPA and nicotine did not produce additional effects of *CHRNA7* in HeLa cells but increased *CHRNA7* expression, and decreased DNA methylation, in SH-SY5Y cells.

Necessary for acetylation of H3K14, dysregulations of bromodomain-containing protein 1 (BRD1), and its encoding gene *BRD1* have been linked to SCZ and BD and show stress-related changes in expression (Christensen et al., 2012; Dyrvig et al., 2017). The effects of CBZ, VPA, and Li on the *BRD1* gene was studied in cultured neuroblastoma cells (SH-SY5Y) providing evidence that these mood stabilizers altered the expression of *BRD1* by mechanisms other than DNA methylation, which remained unchanged after exposure to these agents (Christensen et al., 2012; Dyrvig et al., 2017). Increased expression of total *BRD1* with CBZ was observed, while VPA increased the expression of exon 1C, and Li treatment decreased the expression of exon 1B.

Two papers examined epigenetic changes induced by mood stabilizers on genes involved in synaptic processes, *GABRB2* (encoding a GABA receptor subunit), and *CACNA1C* (encoding a calcium channel) (Zong et al., 2017; Billingsley et al., 2018). GABA_A_ receptor β_2_-subunit gene (*GABRB2)* has been linked to SCZ and BD in multiple studies (Chen et al., 2009; Zhao et al., 2012). Zong et al. (Zong et al., 2017) assessed the response of deregulated *GABRB2* mRNA expression to epigenetic modifications with VPA. Treatment of neuroblastoma cells (IMR-32) with VPA not only induced hyperacetylation of histone H4 and promoter regions, but also had a five-fold increase in mRNA expression providing further evidence of GABAergic dysfunction in psychotic disorders. Billingsley et al. (2018) focused on the effect of lithium and cocaine exposure in the transcriptional activity on *CACNA1C* gene, encoding for the L-type a1c sub-unit of the voltage-dependent calcium channel, Cav1.2, the most abundant human neuronal L-type calcium channel and a significant risk gene for BD and SCZ (Moon et al., 2018; Zhu et al., 2019). Treatment of neuroblastoma cells (SH-SY5Y) with Li resulted in an increased expression of all three studied *CACNA1C* promoters (Billingsley et al., 2018). A gene-specific analysis of DNA methylation status of *BDNF* and *SLC6A4* genes after exposure to VPA, Li, and CBZ showed that all three mood stabilizers decreased, at different concentrations, methylation of CpG sites of *SLC6A4* but not *BDNF* promoter IV in neuroblastoma cells (Asai et al., 2013). This study was limited by the lack of testing of treatment duration but provided, nonetheless, insights into epigenetic regulation exerted by mood stabilizers in two genes involved in synaptic regulation and associated with mood disorders.

Epigenetic variations secondary to VPA in the monoamine system were explored by Manca et al. (Manca et al., 2019) through analysis of *MAOA* gene regulation. Neuroblastoma cells (SH-SY5Y), which is a female cell line, showed a decreased methylation pattern at the uVNTR domain of *MAOA* after exposure to VPA. This study is particularly relevant due to the heterozygous nature of the *MAOA* gene, located on the X chromosome, in females and because findings showed a different pattern of binding of transcription factors on each X chromosome allele.

Two studies provided insights on the influence of mood stabilizers on neuroprotective- and transcription-related genes (Asai et al., 2013; Kao et al., 2013). Activation of *FGF-1B* gene promoter, member of the fibroblast growth factor family, by VPA- and Li-related inhibition of HDACs and GSK-3 pathway in glioblastoma cells was investigated (Kao et al., 2013). VPA-treated cells showed increased expression levels of transcriptional factors *RFX2* and *RFX3*, which bind to *FGF-1B* promoter, as well as activation of *FGF-1B* promoter through acetylation of histone H3 secondary to HDAC inhibition. Through a different pathway, Li, a well-known GSK-3 inhibitor, enhanced the expression levels of *FGF-1B* and *RFX2* (Kao et al., 2013). This study suggests that *FGF1* is an important target of mood stabilizers through different pathways. Asai et al. performed an analysis of the DNA methylation status of *BDNF* and *SLC6A4* after exposure to VPA, CBZ, and Li. Findings suggested a propensity of all three mood stabilizers to alter global DNA methylation and were associated with decreased methylation of *SLC6A4* CpG3 and CpG4. DNA methylation status of BDN promoter IV was not significantly altered by any of the studied stabilizers (Asai et al., 2013).

#### Human Studies

Studies comparing the effects of Li and VPA, alone or in combination with other medications, on DNA methylation of *BDNF* gene of subjects with BD or MDD were similar. A more significant reduction in DNA methylation level was observed with VPA and Li when compared to other medications (D’Addario et al., 2012). Lower methylation levels during manic states observed in this study could be attributed to the use of higher doses of mood stabilizers than during depressive or euthymic states. More recently, this same group generated data suggesting a decrease in DNA methylation level at *BDNF* gene promoter in patients under treatment with VPA or Li, although due to lack of reporting on used doses, a dose-dependent effect on methylation levels remains uncertain (Dell’Osso et al., 2014).

Lithium was also associated with reduced methylation at one site in the 5′ regulatory region of *ARNTL* (cg05733463) when compared to individuals with BD not taking lithium, suggesting an activation of epigenetic marks with an associated increased gene expression (Bengesser et al., 2018). ARNTL, a core component of the circadian clock, is involved in the expression of the *MAOA* gene, therefore imbalances in the relation between circadian rhythms and neurotransmitter degradation may contribute towards an increased BD susceptibility (Geoffroy, 2018). Effects of Li on *FAR2* (fatty acyl CoA reductase 2) methylation and insulin resistance was done in another paper, although the main focus of the research was on second generation antipsychotics (Burghardt et al., 2016). Only 25% of the 72 subjects included were on Li monotherapy, so lack of association between *FAR2* methylation levels and Li treatment could be a consequence of the small sample size. A previous study by Burghardt et al. (Burghardt et al., 2015) also compared global DNA methylation induced second generation antipsychotics versus Li, VPA, and LTG and insulin resistance. No change in global methylation was observed with mood stabilizers but, as in their subsequent study, the small size of the sample under mood stabilizer treatment (31 of 115 BD1 patients) was a significant limitation. Another study measured global DNA methylation and glutathione peroxidase (GPx) activity in subjects with BD and excellent response to Li monotherapy, in unaffected and affected relatives, and in healthy controls (Huzayyin et al., 2014). Interestingly, GPx activity was increased in affected relatives after treatment with Li and a significant, negative correlation between GPx activity and DNA methylation was observed. GPx levels have been reported to be reduced in postmortem prefrontal cortices in SCZ, MDD, and BD (Gawryluk et al., 2011).

Studies indicate that *GAD_67_* mRNA levels are reduced in postmortem brains of subjects diagnosed with SCZ and BD (Akbarian and Huang, 2006). Similar to findings in previously described animal studies, a dual *in vivo* and *in vitro* approach in humans consisting of 4-week treatment with VPA was found to upregulate, in response to dosage, the expression of *GAD_67_* in individuals with SCZ and BD, as well as H3K9 and H3K14 acetylation levels (Gavin et al., 2009). Cultured lymphocytes exposed to 0.7 mM VPA showed a 383% increase in *GAD_67_* mRNA and an 89% increase in H3K9ac and H3K14 while lymphocytes from SCZ and BD patients before and after treatment with VPA showed a significant increase in *GAD_67_* mRNA expression (Gavin et al., 2009). H3K9Ac is an epigenetic mark that typically indicates transcriptionally active chromatin (Qiao et al., 2015), therefore the 482% increase in H3K9ac/H3K14ac attachment to the *GAD_67_* promoter could predict a further increase in *GAD_67_* expression (Gavin et al., 2009).

DNA methylation signatures of psychotropic drugs on candidate-genes in 172 Dutch patients with BD were studied (Houtepen et al., 2016). Impact of the classic mood stabilizers VPA, CBZ, LTG, and Li were measured on *RELN, SLCQA2, MTNR1A, IGF2, H19, BDNF, SLC6A4*, and *GAD1*. No specific methylated CpG sites survived multiple testing correction; Q-Q (quantile-quantile) plot analysis and trend level results suggested that it was likely the result of limited statistical power, although VPA was significantly associated with altered methylation signatures (Houtepen et al., 2016).

It is worth mentioning that all studies on human subjects were performed on peripheral blood mononuclear cells (PBMC), widely used for DNA methylation studies and a model of epigenetic gene regulation in the brain (Gavin and Sharma, 2010; Dell’Osso et al., 2014). Despite DNA methylation being subject to tissue-specific variations in brain and blood, PBMC is considered a reliable biomarker of brain activity (Davies et al., 2012).

#### Studies With Animal Models, Human Cell Lines, and Psychiatric Populations

Two studies performed gene-specific analysis with sample-specific approaches on rodents, human cell lines, and/or patients with BD (Kakiuchi et al., 2003; Kaminsky et al., 2015). Kakiuchi et al. studied the effect of mood stabilizers (Li, VPA, and CBZ) on activating transcription factor gene (*ATF6)*, and heat shock protein family A member 5 gene (*HSPA5)*, on lymphoblastoid cells in two pairs of monozygotic twins affected with BD, a pair of healthy twins, and in neuroblastoma (SHSY5Y) cells (Kakiuchi et al., 2003). Only VPA significantly increased mRNA expression of *ATF6* and induced *HSPA5* gene expression through upregulation of *XBP1* gene. The second study explored the association of mood stabilizers, VPA and Li, with epigenetic changes in *KCNQ3* gene, which encodes for potassium voltage-gated channel, subfamily Q member 3 (Kaminsky et al., 2015). DNA methylation and gene expression levels were tested in four different samples 1) a group comparing post-mortem prefrontal cortex tissue from 12 BD subjects versus 2) 10 control subjects, 3) a sample of human neuroblastoma cell line, and 4) prefrontal cortices of Brown-Norway rats. On the first and fourth groups, increased DNA methylation was observed after treatment with VPA and Li, while no changes were observed in neuroblastoma cells (Kaminsky et al., 2015).

### Risk of Bias

Every article was appraised for quality using a modified published process (du Prel et al., 2009); all papers met a quality score of moderate or high.

## Discussion

### Summary of Main Findings

This systematic review explored the epigenetic changes induced by VPA, Li, CBZ, and LTG in major psychiatric disorders. Despite the genetic complexity of mental illnesses, evidence suggest that epigenome can be targeted by mood stabilizers in psychiatric disorders which can potentially translate into long-term illness trajectory changes (Ludwig and Dwivedi, 2016), although, aside from VPA and Li, the evidence found was limited. Currently, only VPA and Li have shown to consistently induce epigenetic changes while LTG and CBZ have shown less consistent evidence, in part due to the lack of studies or to small sample sizes. Mood stabilizers exert their actions not only through histone deacetylase inhibition, but also through downregulation of methylation of cytosines in CpG dinucleotides, increased histone activity, and induction of RNA, which leads to an augmentation of gene expression (Machado-Vieira et al., 2011; Houston et al., 2013). Previously identified disease-associated alterations in epigenetic markings of specific genes were susceptible to mood stabilizers, according to our findings.

We found few studies primarily focusing on the epigenetic effects of CBZ and LTG. This is relevant because both agents have Level 1 evidence for treatment of different phases of BD and are used in multiple psychiatric disorders (Reid et al., 2013; Yatham et al., 2018). While CBZ has been linked to moderate effects on global and gene-specific DNA methylation and HDAC inhibition, no clear conclusions can be drawn with respect to its epigenetic effects from available literature. Neuroprotective effects of LTG are believed to occur *via* inhibition of glutamate excitotoxicity, HDAC inhibition, histone acetylation, and activity on neuronal survival, and plasticity cascades. Up-regulation of anti-apoptotic gene *Bcl-2* has been studied as a potential mechanisms through which LTG exerts its epigenetic effects (Leng et al., 2013; Reid et al., 2013). It has been previously noted that perturbations in the apoptotic pathway could be involved in the pathogenesis of mood disorders (Zhang et al., 2014; Scaini et al., 2017) and that apoptotic regulator genes are targeted by VPA, Li, and LTG (Leng et al., 2013; Dwivedi and Zhang, 2015). Hence, the therapeutic potential of a synergistic neuroprotection from combined mood stabilizer therapy is a target for future investigation.

VPA is the prototypical HDAC inhibitor in major psychiatric disorders research. VPA relieves class I and II HDAC-dependent repression of transcription factors and increases histones H3 and H4 acetylation thereby enhancing activation of gene expression (Gottlicher et al., 2001). However, evidence suggests that VPA also exerts an effect on DNA methylation signatures, suggesting that VPA’s activity over chromatin remodeling is not limited to HDACs inhibition (Houtepen et al., 2016; Pisanu et al., 2018). It is worth mentioning that HDAC inhibition and associated hyperacetylation by mood stabilizers is not absolute and that increased gene expression is a process that entails high levels of acetylation turnover and recruitment of HATs and HDACs to the active gene, suggesting that HDACs have both inhibitory and activating effects on transcription (Shahbazian and Grunstein, 2007). The latter may explain reports of increased HDAC expression after exposure to mood stabilizers described in several of the included studies (Ookubo et al., 2013; Liu et al., 2014; Bator et al., 2015). Likewise, since DNA methylation and histone modification pathways are not completely independent from each other (Cedar and Bergman, 2009), one mechanism by VPA can partially affect activity in the other. VPA-induced augmentation of methylcytosine-binding protein MeCP2 reported in animal models (Tremolizzo et al., 2002; Kim et al., 2008) suggests a relationship between DNA methylation and histone modification through HDACs recruitment to the methylated region by MeCP2 (Cedar and Bergman, 2009). VPA has also been reported to reduce methylation of gene promoters, thereby upregulating mRNA/expression of genes linked to SCZ and BD, indicating other mechanisms of action additional to HDAC inhibition (Dong et al., 2010). Animal and human studies generally reported that prominent epigenetic activity induced by valproic acid were exerted through different types of epigenetic mechanisms which led to a significant rise in the expression of silenced genes and regulation of altered transcriptional processes (Pisanu et al., 2018).

Although the precise mechanisms through which Li induces neuroprotective and neurotrophic activity are not yet fully elucidated, evidence suggests that activity on synapsis strength, cellular resilience, and glial function at a molecular level has a key role (Machado-Vieira, 2018). Overlapping activity on different pathways is characteristic of Li; however, epigenetic contributions on neurotrophins, apoptosis pathways, and glycogen synthase kinase-3 (GSK-3β) stand out, among the included papers, as central targets of lithium (D’Addario et al., 2012; Kao et al., 2013; Dell’Osso et al., 2014; Dwivedi and Zhang, 2015). Several studies have identified a negative correlation between *Bcl-2* levels and positive symptoms in SCZ and manic symptoms in BD (Tsai et al., 2013; Chen et al., 2015), implying a dysregulation of apoptotic pathways in these disorders. Findings from our search reflect that Li can reverse not only the decreased levels of *Bcl-2* and *Bcl-XL* but also inhibit the expression of pro-apoptotic molecules such as *BAD, BAX* and caspase-3 (Dwivedi and Zhang, 2015), highlighting the anti-apoptotic properties of this mood stabilizer. Similarly, expression of neuroprotective and anti-apoptotic protein *BDNF* has been shown to be downregulated in BD, MDD, and SCZ (Machado-Vieira, 2018) but also epigenetically susceptible to mood stabilizers, including Li which exerts this activity mainly through DNA demethylation (D’Addario et al., 2012; Asai et al., 2013; Dwivedi and Zhang, 2015; Houtepen et al., 2016). Inhibition of GSK-3β activity on the intracellular signaling cascade has been one of the most well-defined mechanisms of action of Li (Beurel et al., 2015). One study reported an upregulation of the expression of plasticity proteins secondary to Li inhibition of GSK-3 activity (Kao et al., 2013). This is an especially important target for future research. Additionally, our systematic process corroborated the already defined role of Li as an HDAC inhibitor (Hobara et al., 2010; Ookubo et al., 2013).

### Strengths and Limitations

While the epigenetic effects of mood stabilizers were evident, there are limitations to our results. First, most studies were limited by relatively small group samples and focused on cohort-based effects. This small group-based approach could hinder the identification of disease-relevant signals and be underpowered (Houston et al., 2013). Although subject to availability of samples and resources, future research should aim to increase the size of the studied groups. Second, studies were heterogeneous in methodology and studied population. Third, the majority of papers were focused on VPA, highlighting an urgent need to study the activity at an epigenetic level of other mood stabilizers. As we previously mentioned, although partly comparable, epigenetic patterns in peripheral blood cells are not an exact reflection of those in brain tissue (Davies et al., 2012; Pisanu et al., 2018), neuroblastoma cells, or animal models; however they are widely accepted as models for research of neuropsychiatric disorders in the absence of cell type-specific epigenome mappings (Houston et al., 2013). Finally, despite the measures taken to reduce publication bias through well-defined inclusion criteria, selective reporting cannot be excluded.

Several strengths were identified in our study. We reviewed a large body of evidence using criteria to capture a specific feature of mood stabilizers in SCZ, BD, and MDD and the quality of scientific publications were evaluated. Since we focused mainly on gene-specific effects of mood stabilizers, we identified small regulatory networks of epigenetic modulators of gene expression and neuroplasticity; consistency across studies was also found. To our knowledge, this systematic review is the first to attempt to summarize epigenetic changes secondary to non-antipsychotic mood stabilizers in three major psychiatric disorders.

## Conclusions

In conclusion, available data confirms the effects of VPA and Li on the epigenome of genes associated with major psychiatric disorders and suggest that LTG and CBZ might have a greater role in the manipulation of epigenetic mechanisms, although further studies are required to elucidate their mechanisms of action. Our systematic review not only reflects the genetic complexity of major psychiatric disorders but, by summarizing the existing evidence, also underscores the ability of these classic mood stabilizers to target genes involved in synaptic plasticity, neuroplasticity, and transcription factors. A better understanding of the specific epigenetic changes induced by classic mood stabilizers in patients with major psychiatric disorders can lead towards more personalized interventions and to the development of new agents able to induce selective chromatin remodeling and gene-specific expression effects.

## Data Availability Statement

The original contributions presented in the study are included in the article/supplementary material, further inquiries can be directed to the corresponding author.

## Author Contributions

MG-R and MV conceived the research idea. MG-R, MV, and CB designed the search strategy. MG-R and MK performed the data collection. AH co-wrote and reviewed the paper. PC, MF, and CB reviewed and edited the paper. MV supervised the research.

## Conflict of Interest

MF’s potential conflicts include previous grant support from Assurex Health, Mayo Foundation, and Medibio; consultancy from Actify Neurotherapies, Allergan, Intra-Cellular Therapies, Inc., Janssen, Myriad, Neuralstem Inc., Takeda, and Teva Pharmaceuticals; CME/Travel/Honoraria from American Physician Institute, CME Outfitters, and Global Academy for Medical Education.

The remaining authors declare that the research was conducted in the absence of any commercial or financial relationships that could be construed as a potential conflict of interest.
